# Pulmonary immune cells and inflammatory cytokine dysregulation are associated with mortality of IL-1R1^-/-^mice infected with influenza virus (H1N1)

**DOI:** 10.24272/j.issn.2095-8137.2017.035

**Published:** 2017-05-18

**Authors:** Lei Guo, Yan-Cui Wang, Jun-Jie Mei, Ruo-Tong Ning, Jing-Jing Wang, Jia-Qi Li, Xi Wang, Hui-Wen Zheng, Hai-Tao Fan, Long-Ding Liu

**Affiliations:** ^1^Department of Viral Immunology, Institute of Medical Biology, Chinese Academy of Medical Science and Peking Union Medical College, Kunming Yunnan 650118, China; ^2^Division of Neonatology, Department of Pediatrics, Children's Hospital of Philadelphia, Perelman School of Medicine, University of Pennsylvania, Philadelphia 19104, USA

**Keywords:** Influenza, Lung inflammation, IL-1 receptor 1, Neutrophil

## Abstract

Respirovirus infection can cause viral pneumonia and acute lung injury (ALI). The interleukin-1 (IL-1) family consists of proinflammatory cytokines that play essential roles in regulating immune and inflammatory responses *in vivo*. IL-1 signaling is associated with protection against respiratory influenza virus infection by mediation of the pulmonary anti-viral immune response and inflammation. We analyzed the infiltration lung immune leukocytes and cytokines that contribute to inflammatory lung pathology and mortality of fatal H1N1 virus-infected IL-1 receptor 1 (IL-1R1) deficient mice. Results showed that early innate immune cells and cytokine/chemokine dysregulation were observed with significantly decreased neutrophil infiltration and IL-6, TNF-α, G-CSF, KC, and MIP-2 cytokine levels in the bronchoalveolar lavage fluid of infected IL-1R1^-/-^ mice in comparison with that of wild type infected mice. The adaptive immune response against the H1N1 virus in IL-1R1^-/-^ mice was impaired with downregulated anti-viral Th1 cell, CD8+ cell, and antibody functions, which contributes to attenuated viral clearance. Histological analysis revealed reduced lung inflammation during early infection but severe lung pathology in late infection in IL-1R1^-/-^ mice compared with that in WT infected mice. Moreover, the infected IL-1R1^-/-^ mice showed markedly reduced neutrophil generation in bone marrow and neutrophil recruitment to the inflamed lung. Together, these results suggest that IL-1 signaling is associated with pulmonary anti-influenza immune response and inflammatory lung injury, particularly via the influence on neutrophil mobilization and inflammatory cytokine/chemokine production.

## INTRODUCTION

Respirovirus infection causes respiratory illness that affects millions of people every year (Coates *et al*., 2015). The virus enters a host through an upper respiratory infection, and directly infects and proliferates in the airway epithelial cells, alveolar epithelial cells, and immune cells. Viral infection triggers cellular immune pathways to express abundant inflammatory cytokines and chemokines, including TNF-α, IL-1α/β, IL-2, IL-4, IL-6, IFN-α/β, CXCL-1/2, CXCL-9/10, MIP-1/2, and MCP-1, in the respiratory tract and alveolar spaces (Kohlmeier & Woodland, 2009; Sanders *et al*., 2011). These cytokines, in turn, recruit innate immune cells such as macrophages, granulocytes, dendritic cells (DCs), and natural killer (NK) cells into the infected lung to exert anti-viral innate immune responses. Following infection, antigen-presenting cells (APCs) migrate back to the lung-draining lymph node and activate adaptive immune T cells and B cells. Thus, the activated cellular and humoral immune responses ultimately clear viral replication and infection (Braciale *et al*., 2012; Tripathi *et al*., 2015). Although an effective anti-viral immune response is necessary for viral clearance, a prolonged or exaggerated response can damage the respiratory tract, endangering respiration physiology. In clinically critical influenza A virus (IAV) infection, mortality is often correlated with severe pneumonia and inflammatory lung injury characterized by massive inflammatory cell infiltration and a cytokine storm in the lung (Damjanovic *et al*., 2012).

Interleukin-1 (IL-1) signaling is accomplished by two major proinflammatory cytokines, namely, IL-1α and IL-1β, both of which bind to the type I IL-1 receptor (IL-1R1) of structural and immune cells in different tissues, leading to transcription and expression of multiple immune/inflammation-associated genes, including cytokines, chemokines, and adhesion molecules (Weber *et al*., 2010). The main function of IL-1 signaling is the regulation of innate immune reactions and inflammatory responses to infections or sterile insults from damaged cells (Dinarello, 2009). Using an IL-1R1 knockout (IL-1R1^-/-^) mouse model, Schmitz et al. revealed that IL-1 plays an essential role in protecting mice from IAV infection and limiting viral proliferation. IL-1 deficiency leads to reduced lung immunopathology at the early infection stage and impairment of the anti-viral immune response (Schmitz *et al*., 2005). To gain insight into the lung immune and inflammation responses regulated by IL-1 signaling from the initial to late infection periods of fatal IAV infection, we continuously monitored lung leukocyte infiltration and cytokine expression in IL-1R1^-/-^ mice infected with a lethal dose of influenza H1N1. Our results suggest that the lack of IL-1 signaling induces an impaired innate and adaptive immune response, together with an increased inflammation response and immunopathology following fatal IAV infection in the infected mouse lung, and contributes to impaired viral clearance and high mortality.

## MATERIALS AND METHODS

### Animals and virus

The IL-1R1^-/-^ and wild type (WT) control C57BL/6 mice were purchased from Jackson Laboratories (USA). The mice were raised and maintained in individually ventilated cages at the Central Animal Care Services of the Institute of Medical Biology, Chinese Academy of Medical Sciences (IMB, CAMS), under specific-pathogen-free conditions. Male mice aged 8-12 weeks were used in all experiments. The mouse-adapted A/Puerto Rico/8/1934 H1N1 (A/PR/8) influenza virus was stored in our laboratory. The virus was grown in chorioallantoic fluid of 9-day-old embryonated chicken eggs. The titer of the virus was determined by 50% cell culture infective dose (CCID_50_) in Madin-Darby canine kidney (MDCK) cells as per Reed and Muench (Szretter *et al*., 2007).

### Mouse infection model

The WT and IL-1R1^-/-^ mice (*n*=35 of each group) were anesthetized by intraperitoneal injection of pentobarbital (50 mg/kg) and then inoculated intranasally with 2 000 CCID_50_ of influenza virus in 30 μL of PBS. Weight loss was monitored and survival curves were generated. All animal experiments were conducted in a biosafety laboratory (BSL-2) with approval from the Animal Ethics Committee of IMB, CAMS, under permit number [2014]43 and in accordance with the National Guidelines on Animal Work in China.

### Viral loads

Mice (*n*=3 of each group) were sacrificed every two days post-infection (dpi), with lung tissue samples then harvested and weighed. Samples were mechanically homogenized using a TGrinder instrument (Tiangen Biotechnologies, China), and RNA was isolated using TRNzol A+ reagent (Tiangen Biotechnologies, China). The RNA concentration of each sample was determined by UV 260/280 using a Nanodrop 2000 (Thermo-Fisher Scientific, USA). Total RNA (100 ng) was reverse transcribed and amplified using the One Step PrimeScript™ RT-PCR Kit (Takara Biotechnologies, China) on an Applied Biosystems 7500 Real-Time PCR System (Life Technologies, USA). To determine the viral loads, the primers and probe for the viral matrix protein gene (M gene) were used: 5'-AAGACCAATCCTGTCACCTCTGA-3' (forward), 5'-CAAAGCGTCTACGCTGC AGTCC-3' (reverse), 5'-(FAM)-TTTGTGTTCACGCTCACCGT-(TAMRA)-3' (probe). The M genes of the A/PR/8 viruses were cloned into the pMD18-T vector, which was used to create a standard curve by 10-fold serial dilution. Viral copy numbers were normalized to the original tissue sample masses and calculated based on the standard curve described above. The RNA samples from each individual were quantified in triplicate.

### BALF cells and total proteins

The mice were anesthetized, sacrificed, and bled (*n*=4 of each group). After the trachea was exposed, the lungs were lavaged four times with 0.8 mL of cold, sterile PBS, and the bronchoalveolar lavage fluid (BALF) was centrifuged at 1 500 g for 10 min at 4 ℃. Supernatants were collected and stored at -80 ℃ for protein, chemokine, and cytokine detection. Total leukocytes in the BALF were counted and resuspended at 10^5^/mL with PBS. The solutions (200 µL) were then cytospun onto slides, and cells were stained with a Wright's-Giemsa stain kit (Nanjing Jiancheng Bioengineering, China) for differential leukocyte counts in five random fields under a microscope.

### Lung pathology and wet/dry weight

Mice were euthanized and the lungs were slowly inflated by instilling 1 mL of 4% formaldehyde intratracheally. The trachea was tightened, and the lungs were fixed in formalin for 48 h at 4 ℃ and paraffin embedded. Lung tissue sections (5 µm) were then stained with hematoxylin and eosin (H & E) according to routine procedures. Images were obtained by light microscopy. Lung histological scores were measured by a blinded pathologist with a 0 to 4-point scale according to combined assessments of alveolar structure, inflammatory cell infiltration, aggregation in alveolar spaces and septa, bronchiolitis, and lung edema. A score of 0 represented no damage; l represented mild damage; 2 represented moderate damage; 3 represented severe damage; and 4 represented very severe histological changes, with an increment of 0.5 if the inflammation fell between two integers. In each tissue sample three random areas were scored and the mean value was calculated. The histology score was the median value of four mice.

The lungs of mice (*n*=3 of each group) were harvested at 9 dpi and weighed immediately (wet weight). The lung tissue was then dried in an oven at 60 ℃ for 72 h and reweighed (dry weight). The wet-to-dry ratio was calculated for each animal to assess tissue edema.

### Hemagglutination inhibition (HI) assay

Sera were prepared from the blood of infected mice (*n*=4 of each group) at 8 dpi. Before the test, any non-specific inhibitors of hemagglutination were removed by diluting sera with receptor destroying enzyme (RDE, Denka Seiken Co., Ltd, Japan) at a 1: 5 ratio, and incubating at 37 ℃ overnight. The enzyme was inactivated by 2 h-incubation at 56 ℃ followed by the addition of 0.1% sodium citrate. The HI assay was performed using the A/PR/8 strain with 1% chicken erythrocytes in V-bottom 96-well microtiter plates.

### Flow cytometry

Bone marrow and mediastinal lymph nodes were aseptically removed at the time of necropsy (*n*=4 of each group). Single-cell suspensions were obtained by flushing the bone marrow or gently pressing the lymph node against a 70-μm strainer into PBS. Cells were washed in fluorescence-activated cell sorting (FACS) buffer after lysing with ammonium chloride potassium (ACK) buffer. Cell surface staining was done using anti-mouse Gr-1-FITC, CD3-PerCP, CD4-PE, CD8-FITC, and CD19-APC (BD Biosciences, USA). For influenza-specific CD8+ cells, mediastinal lymph node cells were stimulated overnight with formalin-inactivated Influenza A PR/8/34 H1N1 (MOI=4). Brefeldin A (BFA, 1 μmol/L) was added after overnight stimulation and cells were incubated for an additional 5 h. After that, cells were surface stained with anti-mouse CD8-FITC, then permeabilized with Cytofix-Cytoperm solution (BD Biosciences, USA) and stained with intracellular anti-mouse IFN-γ-APC. Flow cytometry data were collected on the FACS Canto (BD Biosciences, USA) and analyzed using FlowJo version 7.6.1.

### Cytokine assays

The Bio-Plex Suspension Array System (Bio-Rad, USA) was used to evaluate the BALF cytokines. The mouse cytokine Th1/Th2 assay was introduced to test IL-2, IL-4, IL-5, IL10, IL-12p70, and IFN-γ according to the user manual. The concentrations of cytokines, G-CSF, TNF-α, IL-1β, IL-6, KC, and MIP-2 in the mouse BALF and sera were determined with ELISA kits (Neobioscience, China) in accordance with the manufacturer's instructions.

### Statistical analyses

We performed all statistical analyses with GraphPad Prism software (version 4). The data obtained from all experiments are presented as the means±*SD*, with *P* < 0.05 considered statistically significant using Student's *t*-test.

## RESULTS

### Survival and viral loads in H1N1-infected mouse lungs

**Figure 1:**
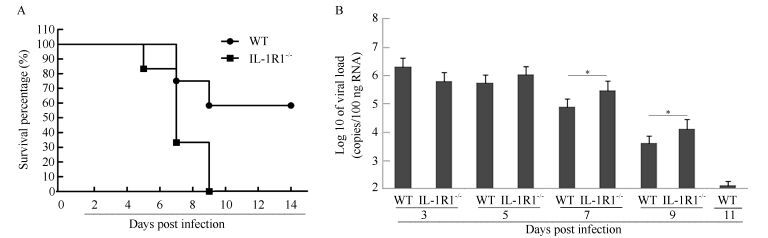
IL-1R1^-/-^ results in enhanced mortality and impaired viral clearance after infection with H1N1 influenza virus

The deaths of WT mice were primarily observed at 7-9 dpi, with no deaths occurring after this time (survival rate 58%) (Figure 1A), whereas the IL-1R1^-/-^ mice began to die at 5 dpi. All IL-1R1^-/-^ mice died by 9 dpi, with no survivors (Figure 1A). Viral replication in the infected mouse lungs was quantified using viral load tests, which reached high levels in both WT and IL-1R1^-/-^ mice at 3 dpi (approximately 10^6^ copies/100 ng RNA) (Figure 1B). The viral loads of WT mice began to decline at 5 dpi, and the virus was cleared to approximately 2×10^2^ copies/100 ng RNA at 11 dpi. In IL-1R1^-/-^ mice, the viral loads began to decrease at 7 dpi, but remained at high levels of 10^5^ and 10^4^ copies/100 ng RNA at 7 and 9 dpi, respectively, which were significantly different than the levels observed in WT mice (Figure 1B). These results revealed that in comparison to WT mice, IL-1R1^-/-^ mice suffered high mortality under fatal H1N1 virus challenge, and failed to effectively clear the H1N1 influenza virus during the progression of infection.

### Histopathology of infected lungs and BALF inflammatory cytokine levels

**Figure 2:**
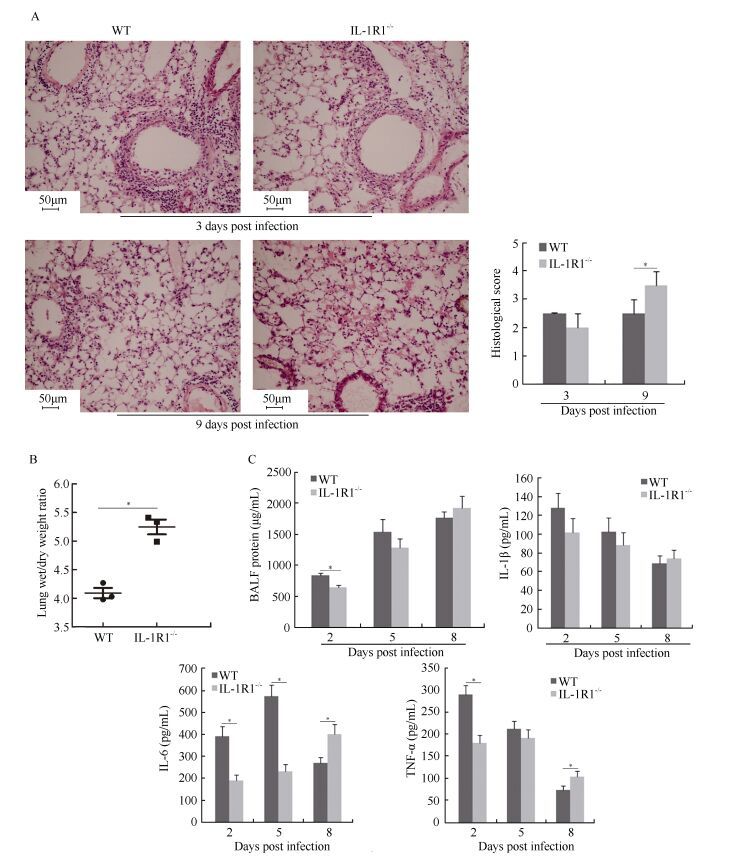
Increased lung inflammation and damage in IL-1R1^-/-^ mice after H1N1 influenza virus infection

The H & E staining of the lung tissue revealed the presence of lung inflammation in both WT and IL-1R1^-/-^ mice at 3 dpi. Symptoms included inflammatory cell infiltration into the lung parenchyma and interstitium, thickening of bronchial epithelium, and alveolar expansion (Figure 2A). Unlike WT mice, IL-1R1^-/-^ mice had less inflammatory cell aggregation in the bronchial walls and alveolar spaces (Figure 2A). At the late stage of infection (9 dpi), lung inflammation had gradually regressed in the surviving WT mice, with the alveolar structure tending to be complete and a small number of inflammatory cells aggregating around a portion of the lung parenchyma (Figure 2A). However, both disruption of the alveolar structure and formation of a hyaline membrane were seen in the lungs of dying IL-1R1^-/-^ mice during late infection, with congestion and swelling of the lung interstitium (Figure 2A). Histological scores demonstrated that IL-1R1^-/-^ mice exhibited less severe lung pathology than that of WT mice during the early infection stage, but significantly more severe lung pathology during the late infection stage (Figure 2A). The lung wet-to-dry ratios of IL-1R1^-/-^ mice were significantly higher than those of WT mice at 9 dpi, suggesting that the IL-1R1^-/-^ mice suffered more severe lung edema during late infection (Figure 2B). Analysis of the total protein concentrations and inflammatory cytokine levels in the BALF from infected mice showed that the protein concentration and IL-1β, IL-6, and TNF-α levels were significantly higher in WT mice than in IL-1R1^-/-^ mice at 2 dpi (Figure 2C). At 8 dpi, the levels of IL-6 and TNF-α were significantly higher in IL-1R1^-/-^ mice than in WT mice (Figure 2C). Thus, compared with the decreasing inflammation and pathology in the lungs of WT mice, the inflammatory lung pathology was continuously aggravated in IL-1R1^-/-^ mice.

### BALF leukocytes and viral-induced immune response analysis

**Figure 3:**
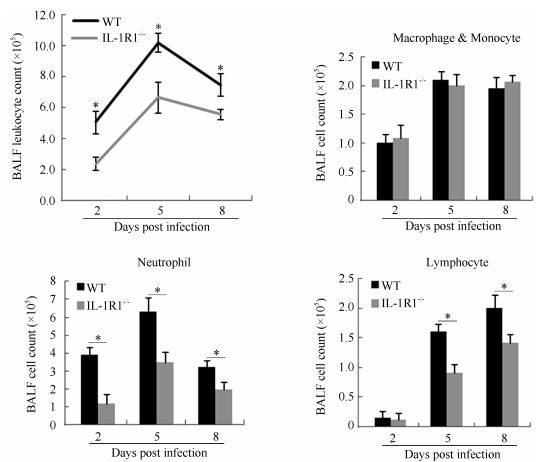
Alteration in accumulation of leukocytes in the lung BALF of IL-1R1^-/-^ mice after H1N1 influenza virus infection

**Figure 4:**
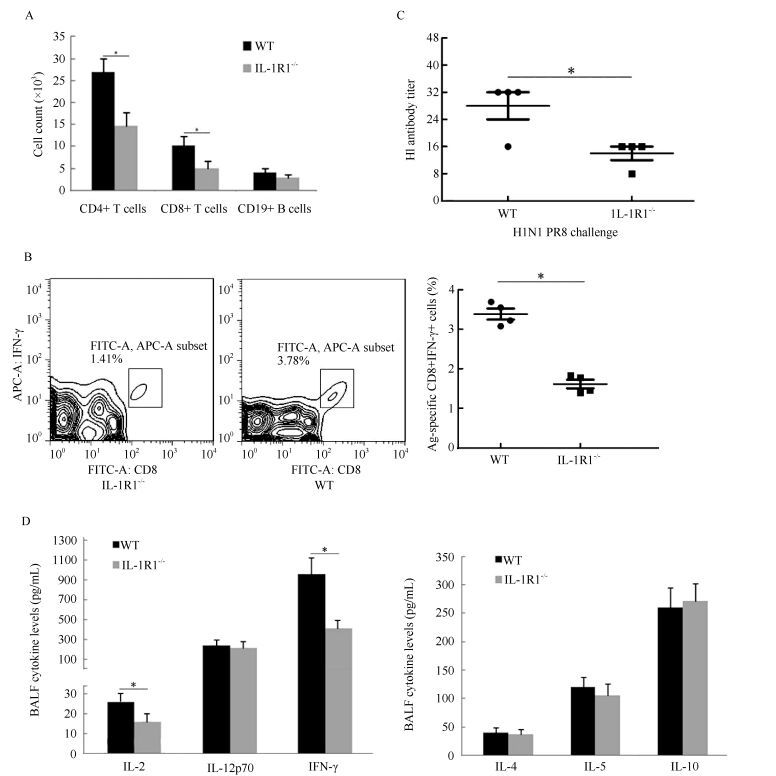
Impaired adaptive immune response in the lung of H1N1 infected IL-1R1^-/-^ mice

Lung BALF leukocytes from infected WT and IL-1R1^-/-^ mice were continuously monitored. Analysis of total leukocyte numbers in the BALF of both WT and IL-1R1^-/-^ mice showed that the number of leukocytes increased sharply during initial infection (2-5 dpi) and peaked at 5 dpi before decreasing gradually (Figure 3). In IL-1R1^-/-^ mice, however, the numbers of leukocytes were significantly lower than those of WT mice at every post-infection time point. Cell smearing and counting for different leukocytes in the lung BALF were performed. Results showed that during infection, the numbers of neutrophils were significantly lower in IL-1R1^-/-^ mice than in WT mice, whereas the numbers of macrophages and monocytes tended to be the same (Figure 3). The BALF lymphocytes increased following infection, and the numbers of lymphocytes in IL-1R1^-/-^ mice were significantly lower than that in WT mice at the late stage of infection (5-8 dpi) (Figure 3). Further, viral-induced T, B lymphocyte immune responses were analyzed. The numbers of BALF CD4+, CD8+, and CD19+ lymphocytes were lower in IL-1R1^-/-^ mice than in WT mice, with statistically significant differences in CD4+ and CD8+ T lymphocytes (Figure 4A). Lymphocytes from the lung-draining mediastinal lymph nodes of infected mice were isolated and challenged with the H1N1 virus, with subsequent flow cytometry revealing that the percentages of CD8 and IFN-γ double-positive cells in IL-1R1^-/-^ mice were significantly lower than that in WT mice (Figure 4B). In addition, HI assay revealed a significant decrease in specific antibodies against H1N1 in the sera of infected IL-1R1^-/-^ mice compared with that of infected WT mice (Figure 4C). We used the Bio-Plex Mouse Cytokine Th1/Th2 assay to analyze Th1 and Th2 cytokines in the lung BALF of infected mice. Although the levels of Th2 cytokines were almost the same in both groups of mice, the expression levels of Th1 cytokines IL-2 and IFN-γ were significantly lower in IL-1R1 knockout mice than in WT mice (Figure 4D). Collectively, these data suggest that IL-1R1 deficiency in mice results in an impairment of the anti-viral immune response in infected lungs, with decreased neutrophil accumulation and downregulated T and B lymphocyte immune responses.

### Analysis of neutrophils in blood and bone marrow and G-CSF, KC, and MIP-2 levels in lung BALF and serum

**Figure 5:**
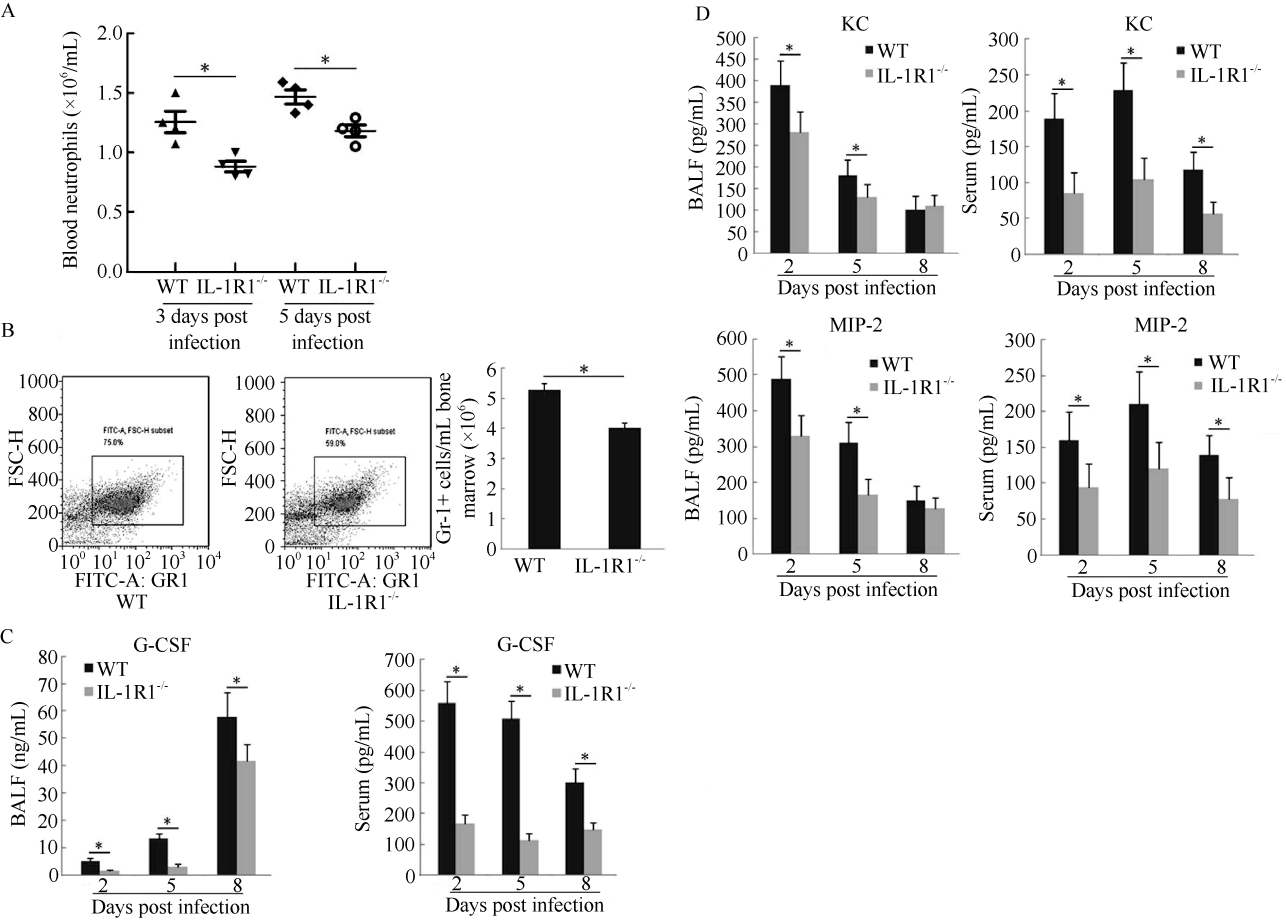
IL-1R1^-/-^ interferes with neutrophil generation and mobilization to the H1N1-infected mouse lung

Neutrophils are generated in the bone marrow and released into peripheral blood circulation to monitor the immune status of the body. When a peripheral organ such as the lung releases inflammatory signals, activated neutrophils are recruited from the vasculature to the inflammatory tissue to exert their functions (Kolaczkowska & Kubes, 2013). To determine whether the decrease in neutrophils in lung BALF from infected IL-1R1^-/-^ mice was caused by impeded neutrophil recruitment and/or neutrophil generation, numbers of neutrophils in the peripheral blood and bone marrow were analyzed. Results showed that IL-1R1^-/-^ mice had significantly fewer neutrophils than WT mice at 3 and 5 dpi in serum (Figure 5A). In the bone marrow, the Gr-1+ cell population of IL-1R1^-/-^ mice was significantly lower than that of WT mice at 5 dpi (Figure 5B). The cytokine G-CSF plays an important role in the generation and maturation of bone marrow neutrophils (Bendall & Bradstock, 2014). The levels of G-CSF in the serum and lung BALF of IL-1R1^-/-^ mice were lower than that of WT mice during infection (Figure 5C). The above results indicate that IL-1R1 is essential for the generation of neutrophils in the bone marrow. In addition, mouse chemokines KC and MIP-2 play major roles in neutrophil recruitment to inflammatory tissues (Olson & Ley, 2002). Analysis of the lung BALF and serum KC and MIP-2 levels showed that both chemokines were significantly lower in IL-1R1^-/-^ mice than in WT mice, especially at 2 and 5 dpi (Figure 5D). These results reveal that knocking out IL-1R1 inhibited neutrophil generation and recruitment to H1N1-infected mouse lungs.

## DISCUSSION

Understanding the contributions of IAV-infected lung pathology over the course of an acute infection remains a puzzle because distinct IAV infection and proliferation in the respiratory system can cause direct viral injury and indirect immunolesions with activated innate and adaptive anti-viral immune responses. IL-1, which consists of two major proinflammatory cytokines (IL-1α and IL-1β), is highly expressed in respiratory cells upon pathogen invasion (Strieter *et al*., 2003). IL-1α and IL-1β bind to IL-1R1, leading to the activation of IL-1 signal-regulated innate immune and inflammatory physiological functions. The role of IL-1R1 signaling in the morbidity and mortality of IAV infection was previously investigated (Schmitz *et al*., 2005), and our results confirm the importance of IL-1R1 signaling in protecting mice against fatal IAV infection and moderating the inhibition of viral replication. In the previous work, the inflammatory lung pathology of WT mice was more severe than that of IL-1R1^-/-^ mice at the early infection stage (3 dpi). Our work also indicated that IL-1R1^-/-^ mice had less severe lung inflammation pathology than that of WT mice at the same early infection stage. However, at the late stage of infection (9 dpi), while lung inflammation of WT mice had gradually regressed, lung pathology in IL-1R1^-/-^ mice was aggravated and resulted in lung damage and edema, characterized by alveolar structure disruption and hyaline membrane formation in the lung parenchyma as well as congestion and swelling of the lung interstitium. Quantitative analysis of lung histological changes, determination of BALF protein concentration, and measurement of proinflammatory cytokines IL-1β, IL-6, and TNF-α indicated that the loss of IL-1R1 signaling reduced lung inflammation at the beginning of IAV infection, but contributed to lung inflammatory injury following infection. This is similar to the roles of critical proinflammatory cytokines TNF-α and IL-6, with the absence of either protein resulting in severe lung inflammation and immunopathology in mice, as well as altered inflammatory cell infiltration at late stage respiratory influenza infection (Damjanovic *et al*., 2011; Dienz *et al*., 2012). Considering the limited inhibition of viral replication that occurs through IL-1R1 signaling, it seems that inflammatory injury plays a more important role in influenza-induced acute lung injury than direct viral proliferation effects upon respiratory H1N1 infection.

Decreased innate cellular influx into the airways of IL-1R1^-/-^ mice was observed in the early infection period, which was primarily due to the significantly reduced neutrophil infiltration into the infected lungs, based on the BALF cell count. Among the first immune cells to arrive at a site of infection, neutrophils exert innate immune anti-IAV effects by eliminating infected cells and clearing the virus, dead cells, and debris (Mantovani *et al*., 2011). In addition to their phagocytic and cytotoxic activities, neutrophils facilitate the adaptive immune response against invasive pathogens by serving as APCs, influencing the maturation of DCs, promoting T cell responses, and supporting B cell activity (Leliefeld *et al*., 2015). Our results revealed that the loss of IL-1R1 signaling downregulated the anti-IAV adaptive immune response in the infected lung with decreased BALF T and B lymphocyte numbers, IAV-specific CD8 cytotoxic T cells, and serum antibodies. We also found that IL-1R1 signaling played an important role in the Th1 and Th2 immune response. However, the role of IL-1 signaling in the development of Th1 and Th2 responses is controversial. Some studies have suggested that IL-1 is responsible for the Th2 response (Greenbaum *et al*., 1988; Lichtman *et al*., 1988; Satoskar *et al*., 1998), whereas others indicate that it is involved in regulating the Th1 response (Durrant *et al*., 2013; O'Garra & Murphy, 1994). These variations might be due to the different pathogens and insults used, and differences between *in vitro* and *in vivo* experiments. Our data suggest that IL-1R1 signaling is required for Th1 responses, but is not essential for the generation of Th2 responses in the local IAV-infected mouse lung. Together, loss of IL-1R1 impaired the IAV-specific adaptive immune response by directly blocking the proinflammatory IL-1 signal, and possibly by the indirect effect of downregulating neutrophils that function in activating adaptive immunity.

Our findings of decreased neutrophils in the BALF, peripheral blood, and bone marrow of IAV-infected IL-1R1^-/-^ mice suggests that IL-1 signaling influences neutrophil homeostasis. Indeed, the downstream consequences of IL-1 pathway activation include the upregulation of a cascade of inflammatory mediators. This includes the increased expression of cytokines and chemokines such as IL-6, TNF-α, G-CSF, CXCL1, and CXCL2 (Besnard *et al*., 2012; Dinarello, 2009). G-CSF stimulates the production and maturation of neutrophils by promoting the proliferation and differentiation of myeloid progenitors. In addition, G-CSF promotes neutrophil release from the bone marrow into circulation (Bendall & Bradstock, 2014; Furze & Rankin, 2008). In the present study, we found lower concentrations of the G-CSF protein in both the BALF and serum of IL-1R1^-/-^ mice. Thus, IL-1 signaling possibly affects neutrophil generation in the bone marrow of IAV-infected mice via the G-CSF pathway. Both CXCL1 and CXCL2 (murine KC and MIP-2) have previously been suggested as the two most important chemokines for neutrophil mobilization from bone marrow and recruitment into the lung in rodents (Olson & Ley, 2002; Strydom & Rankin, 2013). Our results also demonstrated that the expressions of CXCL1 and CXCL2 were reduced at the local (infected lung) and system (serum) level in IL-1R1^-/-^ mice. Therefore, based on our results, IL-1 signaling might influence neutrophil homeostasis through neutrophil generation and mobilization, thus contributing to anti-viral neutrophil physiology.

Our results showed that IL-1 signaling is critical to pulmonary anti-influenza immune responses and lung immunopathology, likely due to its effects on regulating innate immune cell infiltration and inflammatory cytokine/chemokine production, especially that of neutrophils. Neutrophils are essential innate immune and inflammatory cells during respiratory influenza infection, and IL-1 signaling might mediate neutrophil generation and recruitment to the infected mouse lung through the G-CSF and CXCL1/2 pathways.
